# Is There Any Correlation Between Green Synthesis Parameters and the Properties of Obtained Selenium Nanoparticles?

**DOI:** 10.3390/molecules30132865

**Published:** 2025-07-05

**Authors:** Aleksandra Sentkowska, Julia Folcik, Jakub Szmytke, Anna Grudniak

**Affiliations:** 1 Heavy Ion Laboratory, University of Warsaw, Pasteura 5A, 02-093 Warsaw, Poland; 2 Department of Bacterial Genetics, Institute of Microbiology, Faculty of Biology, University of Warsaw, Miecznikowa 1, 02-096 Warsaw, Poland; j.folcik@student.uw.edu.pl (J.F.); j.szmytke@student.uw.edu.pl (J.S.); a.grudniak@uw.edu.pl (A.G.)

**Keywords:** selenium nanoparticles, green synthesis, herbal extracts, antioxidant activities, antibacterial activity

## Abstract

Selenium nanoparticles (SeNPs) show enormous potential in biomedical applications. In recent years, green methods of their synthesis have become very popular. In this work, the influence of green synthesis conditions on the properties of the obtained nanoparticles was investigated. For this purpose, extracts of eight medicinal herbs were used, and the reaction was carried out with changing ratios of reagents and variable temperature. All obtained SeNPs were characterized by high stability, which is confirmed by the negative values of their zeta potential ranging from −11.8 to −29.4 mV. The highest correlation coefficient was determined between the size of the obtained SeNPs and the ratio of reagents used for the synthesis (the correlation coefficient is 0.681 for the synthesis carried out at room temperature and 0.914 for elevated temperature). In each case, the smallest nanoparticles were obtained from the synthesis carried out in a 1:1 reagent ratio. It was assessed that sometimes it is difficult to determine correlations between the results collected for all syntheses; therefore, the same correlations determined for specific herbs were also analyzed.

## 1. Introduction

In recent decades, nanomedicine has undergone intensive development. Nanomedicine is an innovative application of nanotechnology in many aspects of medicine, including the early detection, diagnosis, and follow-up of a wide range of serious diseases, including cancer [1]. Many scientists believe this branch of science is crucial to developing a new generation of drugs and therapies, often personalized, which would be a real medical breakthrough. The unique features of nanomaterials, such as their limited size, biocompatibility, and ability to transport potential drugs across cell membranes, make them key to the development of nanomedicine. It should therefore come as no surprise that, in recent years, there has been an increasing interest in nanoparticles (NPs). Recent studies have confirmed the enormous potential of nanoparticles as drug carriers, allowing the delivery of therapeutic agents directly to the affected area [2,3].

Numerous studies have proved the potential of selenium nanoparticles (SeNPs) in some medicinal applications, including drug delivery carriers, antioxidants, and antibacterial and anti-inflammatory effects [4,5]. Several chemical and physical methodologies for SeNP synthesis have been described; however, eco-friendly, green methods have been favored lately. SeNP synthesis is based on nontoxic solvents, in which reducing agents are easily accessible, cheap, and biodegradable. However, this approach also has drawbacks that have been widely commented on. It seems more difficult to control the course of the reaction and transfer the synthesis from laboratory scale to industrial scale. Plant-derived compounds such as flavonoids and polyphenolic acids can act as selenium reducers during the synthesis and stabilization of obtained nanoparticles, so the green synthesis of SeNPs involving plant extracts seems to be a promising perspective in the pharmacological use of SeNPs. However, it is necessary to take into account the fact that the polyphenol profile of a given plant depends on the conditions of its cultivation [6]. Therefore, SeNPs obtained in two parallel syntheses using extracts from plants of the same species but from different crops may differ in their properties. Currently, chemical methods are more suitable for industrial scaling because of their reliability and possibility to control parameters. This is why work on new chemical syntheses is ongoing, and new procedures for obtaining SeNPs are being published. Blinov et al. developed the synthesis of SeNPS stabilized by alkyldimethylbenzylamonium chloride [7]. The method described is characterized by execution simplicity and high reproducibility. The authors emphasize its potential in the cosmetics and pharmaceutical industries. However, they do not describe the procedure for the purification of the obtained SeNPs, which has a significant impact on the properties of the obtained nanoparticles [8]. Chemical synthesis entails the need to purify SeNPs from the post-reaction mixture, which may contain toxic substances. On the other hand, the purification step in the green synthesis of SeNPs is not free of difficulties. For example, the isolation of microorganisms used during the biological synthesis of SeNPs is complicated and significantly increases the cost of the entire procedure [9]. Sarkar et al. carried out a successful green synthesis of SeNPs using the pathogen Alternaria alternata [10]. This approach resulted in the formation of monodisperse spherical α-selenium nanoparticles in the range of 30–150 nm. In this case, nanoparticles were separated from the post-reaction mixture using centrifugation at 12,000× *g* for 10 min, and then settled SeNPs were washed three times in deionized water. Such an easy purification step is impossible to be applied in green synthesis using plant extracts. In this case, SeNPs are so strongly stabilized by the plant extract that difficulties arise with the isolation of SeNPs from the post-reaction mixture [11]. This phenomenon is quite surprising because, in aqueous media and in the absence of stabilizers, selenium nanoparticles are prone to aggregate [12,13]. In summary, the current literature shows that each approach to the synthesis of SeNPs is not free from limitations and drawbacks. Therefore, new procedures for their preparation are being developed, both chemical and green.

Our study tries to answer the key question of how the green synthesis of SeNPs affects their physical properties such as size and homogeneity and, therefore, their antibacterial and antioxidant properties. This is a summary of our research based on extracts of well-known herbs with medicinal properties, including yarrow (*Achillea* L.), blackberry (*Rubus* L.), sage (*Salvia officinalis* L.), nettle (*Urtica* L.), hop (*Humulus* L.), lemon balm (*Melissa officinalis* L.), Ribwort plantatin (*Plantago lanceolata* L.), and raspberry (*Rubus idaeus* L.). Syntheses were carried out at different ratios of reagents at elevated temperatures. The obtained SeNPs were measured for their size and homogeneity, their ability to neutralize DPPH and OH radicals, and their reducing properties using the CUPRAC method, and the antioxidant properties of their suspension were determined based on the Folin–Ciocalteu method. Furthermore, the antibacterial properties of the obtained SeNPs were tested by determining the minimum inhibitor concentration (MIC) against two model bacterial species: *Escherichia coli* (Gram-negative bacterium) and *Staphylococcus aureus* (Gram-positive bacterium). Finally, we attempted to correlate the obtained results with the conditions of green synthesis, which may be valuable for other research groups working with similar methods of obtaining nanoparticles, not only selenium.

## 2. Results and Discussion

### 2.1. General Observations and Overall Correlations

The green synthesis of SeNPs was performed using eight different herbal extracts. Bioactive compounds such as polyphenols, flavonoids, alkaloids, polysaccharides, etc., present in the plant extract could act both as reducing agents and stabilizers. However, the interactions between polyphenolic compounds and nanoparticles are bidirectional. Green synthesized NPs can improve the water solubility, stability, and bioavailability of polyphenols using a different biology-based nano-delivery system [13]. Nanoparticles can also construct complexes with different polyphenolic substances, thereby altering their bioavailability and functional properties. Zhang et al. strongly emphasize that the polyphenol delivery system based on polymerized nanoparticles is a potential solution with which to enhance their absorption in the gastrointestinal tract, improve their bioavailability, and deliver them to target organs [13]. Liu et al. also reached similar conclusions, presenting the potential biological applications of the nano-system to enhance the antibacterial ability of polyphenols, aiming to provide a new therapeutic perspective for the antibiotic-free treatment of infectious diseases [14]. In general, interactions between bioactive compounds and nanoparticles can also have a great impact on the food industry [15]. For example, in the experiment performed by Sari et al., nano-encapsuled curcumin isolated from turmeric (*Curcuma longa*) showed a decreased amount of antioxidant activity and remained resistant after pasteurization [16]. On the other hand, it should be noted that similar interactions take place in the post-reaction mixture after the green synthesis of SeNPs. These, in turn, can affect the properties of the obtained SeNPs and falsify the correlations studied [17].

Each extract used differs in its polyphenol profile and content of other chemical compounds, e.g., ascorbic acid, as reported partially in our previous work [18]. Detailed results of the qualitative and quantitative analysis of the polyphenolic compounds present in the tested extracts are summarized in Table S1.

**Figure 1:**
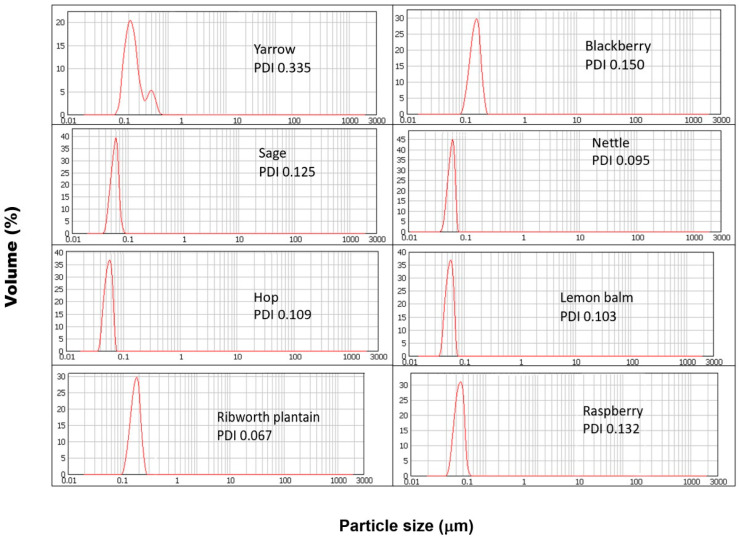
DLS results obtained for SeNPs synthesized using reagents in a 1:1 ratio.

It should therefore be assumed that nanoparticles obtained in each of the parallel syntheses will differ in their physical parameters and properties. Among the properties determined, most of them include the morphology, particle size, zeta potential, and homogeneity. The obtained results and the physical parameters of the synthesized SeNPs are presented in Table 1. The determination of nanoparticle size is essential. In the case of systems for medicinal and biomedical applications, the diameter of the particles will determine the cell penetration properties [19]. The DLS results obtained for the SeNPs synthesized using reagents in a 1:1 ratio are presented in Figure 1. The obtained results show that in each case, increasing the extract to the selenium ratio results in an increase in the size of the obtained SeNPs. These observations are confirmed by high correlation coefficients between the dimensions of the obtained SeNPs and the ratio of the reactants. For the synthesis carried out at room temperature, the correlation coefficient is 0.681, while in the case of additionally heated SeNPs, the value is significantly higher and equal to 0.914. According to the literature, the smaller the dimensions of nanoparticles, the higher their antioxidant properties [20,21]. The results obtained do not confirm these reports. The determined correlation coefficients between the dimensions of the obtained SeNPs and their antioxidant properties determined by all four methods (Folin–Ciocalteu assay, CUPRAC method, DPPH scavenging, and hydroxyl radical scavenging) as well as the size of the SeNPs and the total antioxidant coefficient are low for both temperature variants of the syntheses. Results reported by Sentkowska and Pyrzyńska indicated that the antioxidant activity of SeNPs does not always depend only on the nanoparticles’ size but also on their homogeneity [8]. However, in this case, our results do not confirm this theory. The correlation coefficients between the PDI values and antioxidant capacity values of SeNPs are low. When the concentration of the extract used to reduce selenium salts is increased, nanoparticles of larger dimensions are obtained. At the same time, with the increase in their size, their homogeneity increases, which is illustrated by the decreasing PDI value. According to the theory, the higher the PDI value, the larger the particle size distribution in the analyzed sample [22]. A sample is considered monodisperse when the PDI value is less than 0.1. The correlation coefficients collected in Table 2A,B show that the greatest influence on the properties of the obtained SeNPs is the ratio of reagents in which the synthesis is carried out. The correlation coefficient between nanoparticle size and reactant ratio is high and equal to 0.937 for syntheses carried out without heating and 0.914 for those carried out with sample incubation. The smallest nanoparticles were obtained from the synthesis carried out for a 1:1 reagent ratio, regardless of the type of extract used. Carrying out the synthesis at elevated temperatures is recommended to obtain more homogeneous nanoparticles [23]. Other authors reported that the heating of SeNPs caused their aggregation to a larger size and rods, which resulted in a reduction in their bioactivity [21]. Our results show that there is no clear correlation between the heating and size of obtained nanoparticles. Maybe it is because of the small difference between room and elevated temperature. It should be noted that in the case of our synthesis, the increased temperature was 70 °C. It was not decided to increase it due to the instability of some polyphenolic compounds at high temperatures [24]. These compounds are key to the course of the entire synthesis. The authors of the mentioned publications conducted chemical synthesis, hence the possibility of using higher temperatures, e.g., 90–150 °C, in case of synthesis involving chitosan [25]. The authors reported that the particle sizes were about 83, 142, and 208 nm at the reaction temperatures of 90, 120, and 150 °C; therefore, higher temperature favors the formation of larger nanoparticles. In the case of our results, the size of the obtained SeNPs was in range of 94–182 nm for synthesis performed at elevated temperatures. Some differences in the reaction mechanism between chemical and green synthesis are also possible. Melinas et al. performed SeNP synthesis using *Theobroma cacao* L. extract [26]. To increase the reaction kinetics, microwave irradiation and rapid heating were used. However, the final conclusion of the authors was that neither heating nor microwaves affect the size of the obtained nanoparticles. It was found that the size of SeNPs is influenced only by the concentration of the selenium precursor. On the other hand, Fardsadegh and Jafarizadeh-Malmiri found that at a constant amount of Na_2_SeO_3_, an increase in the amount of Aloe vera extract affected the SeNPs concentration [27]. In the opposite situation, i.e., at a constant extract concentration and increasing selenium precursor concentration, a decrease in the concentration of obtained nanoparticles was observed. However, in the case of our experiment, the correlation coefficient between the value of PDI and reagent ratio was much higher for the syntheses, which were carried out at higher temperatures (0.880 in comparison to 0.681, for those performed at room temperature). Surprisingly, no correlation was observed between the size of the nanoparticles and the antioxidant and antibacterial capacity of the obtained nanoparticles. It is possible that not only size but also other physical properties are responsible for these properties. However, Boroumand showed that SeNPs exhibit dose-dependent activity against all tested bacterial strains [28]. Thus, the inhibitory effect of SeNPs on bacteria may be due to their sorption on the bacteria cell wall, penetration through membrane, and entrance to kill the cell [29]. In such situations, the size of SeNPs is crucial when the mechanism of their action is mainly based on their action inside the cell. SeNPs with a smaller diameter may diffuse bacteria cells easier in comparison to those with larger sizes. In this case, the coefficients for both synthesis variants (with and without heating) were low. Similar observations were made by examining the value of the relationship between the PDI coefficient and the antioxidant and antibacterial capacity of the obtained SeNPs. This contradicts studies previously described, which suggested a significant influence of the homogeneity of the tested SeNPs on their antioxidant properties. However, these studies concerned their chemical synthesis [8]. The chemical approach is characterized by complete control over the concentration and type of reagents; here, a plant extract containing many chemical compounds in varying concentrations is used. Furthermore, the same plant species, but grown under different conditions, will have a different polyphenol profile [30]. This may be another complication in the repeatability and reproducibility of the developed green nanoparticle syntheses. The obtained results indicate a high correlation between the ratio of reagents and the ability of the obtained selenium nanoparticles to neutralize DPPH radicals. In the case of synthesis with heating, this tendency is proportional to the increasing concentration of the extract used for the reaction (the correlation coefficient is 0.964). The analogous coefficient determined for the synthesis carried out at room temperature suggests an inversely proportional tendency (a correlation coefficient equal to −0.850). On the other hand, only a moderate correlation was obtained between the hydroxyl radical scavenging capacity and the reactant ratio for synthesis at room temperature, where the corresponding correlation coefficient was 0.659. The correlation coefficient for the synthesis carried out at elevated temperatures was half as high (0.362). Similar correlation coefficients were obtained for both synthesis variants for the dependence of the reactant ratio on the total polyphenol content in the reaction mixture determined using the Folin–Ciocalteu method (0.693 and 0.716, respectively). The approach to green synthesis involves secondary plant metabolites, including polyphenolic compounds, both natural reductors of selenium salt and stabilizers of the obtained SeNPs. According to Marslin, the mechanism of their action in the synthesis of NPs is complex, and heating plays a key role in providing energy to the reaction system [31]. At the same time, there is a moderate correlation between the reducing properties of the nanoparticle suspensions derived from the room temperature synthesis determined by the CUPRAC method and the reactant ratio (the correlation coefficient is 0.522). There is no analogous correlation for the synthesis carried out at elevated temperatures (correlation coefficient, 0.031). However, the AOX correlation indices determined on the results obtained from all four methods were surprisingly similar and amounted to −0.998 and −0.996, suggesting an inverse correlation between AOX and the reactant ratio. At the same time, no correlation was found between the antibacterial properties, size, and homogeneity of the SeNPs obtained through both synthesis variants. Only the minimum inhibitor concentration (MIC) against *Escherichia coli* and the reactant ratio was high in both syntheses, with the difference being that for synthesis at room temperature, it was inversely proportional, and for elevated temperature, it was proportional (the value was the same and equal: 0.850). The zeta potential of nanoparticles is an electrokinetic measure that describes the potential difference between the surface of a nanoparticle and the surrounding solution [32]. This charge of the SeNPs can be interpreted as an electrical barrier that impedes the coalescence of the particles, thereby helping to stabilize the suspension [33,34]. A high zeta potential (usually above ±30 mV) indicates dispersion stability because nanoparticles with similar charges repel each other, preventing agglomeration [35]. According to dos Santos Souza et al., ζ-potential values in the range of ±0–10 mV indicate that SeNPs are very unstable; those in the range of ±10–20 mV indicate little stability, and those of ±20–30 mV indicate the SeNPs can be stable and very stable [36]. All synthesized SeNPs had zeta potential between −11.8 and −29.4 mV, indicating little stability and few stable nanoparticles in solution. The highest correlation coefficient value was obtained for the zeta potential and reactant ratio. These trends were observed for both room and elevated temperature synthesis (0.936 and 0.784, respectively). As the concentration of the extract used for synthesis increases, the zeta potential value increases. Increasing the extract concentration increases the concentration of polyphenolic compounds in the post-reaction mixture, and they play the role of stabilizers in the green synthesis of SeNPs. However, no correlation was found between the zeta potential value and other properties of the obtained nanoparticles. Zhang et al. obtained SeNPs with an average size of 120 nm and −25 mV of ζ-potential using *Providencia* sp. DCX [37]. Sarkar et al. link the negative value of the zeta potential with their high stability without a tendency to aggregate [10]. The authors point out that nanoparticles do not tend to transform into a black amorphous form when kept for more than a month. The same conclusions were reached by Cavalu et al., who report a zeta potential of SeNPs equal to –38.2 mV, while the maximum percentage of size was set in the range of 20–40 nm [38].

### 2.2. Detailed Correlations Determined for Each Herb

**Figure 2:**
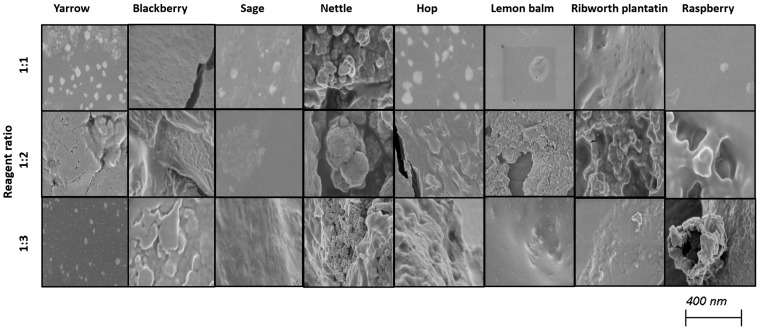
SEM images of obtained SeNPs.

**Figure 3:**
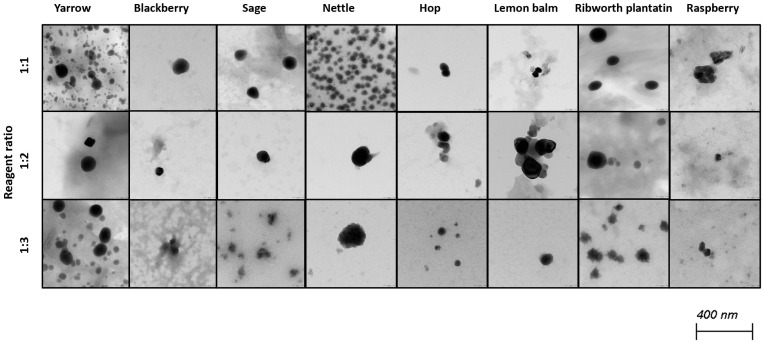
TEM images of obtained SeNPs.

Selenium nanoparticles have been synthesized in various forms such as nanowires, nanorods, and nanotubes [39]. Figure 2 and Figure 3 illustrate TEM and SEM images of SeNPs obtained at room temperature.

These results confirm that more spherical-shaped and smaller nanoparticles were obtained in syntheses where the ratio of reactants is 1 to 1. According to Chen et al., increasing the temperature of the synthesis affects the size and the shape of SeNPs; however, our study did not confirm these findings [25]. Additional heating did not influence the shape of obtained NPs. Different syntheses described in the literature result in different shapes of the obtained selenium nanoparticles. Fritea et al. reported receiving a mixture of nanospheres and nanorods, obtained from the synthesis involving rispum (parsley) leaf extracts and NaHSeO_3_ [40]. On the other hand, spherical SeNPs with a diameter of 10–50 nm or in the range 50–80 nm, or spherical with a maximum number of particles with a size around 400 nm, were also reported [41,42,43]. It should be assumed that the size and morphology of SeNPs obtained using plant extracts depend strictly on their polyphenolic profile. Ramamurthy et al. claimed that SeNPs obtained through biological methods were slightly bigger than those achieved through chemical method [44]. Each herb used for the syntheses that we described differs in its polyphenolic profile, which we partially reported earlier [14]. This may cause the correlations determined above to be inaccurate, which is why we decided to determine analogous relationships for each plant separately, as summarized in Table 3. A high correlation was obtained between the size of the obtained SeNPs and the ability to neutralize free radicals for sage, lemon balm and raspberry (OH assay), nettle and ribwort plantain (DPPH assay), and hop (both assays). Stable 2,2-diphenyl-1-picrylhydrazyl radicals (DPPH) are widely used to evaluate antioxidant activity for a wide variety of samples, also for nanoparticles; however, from the medicinal point of view, the scavenging of hydroxyl radicals is much more important due to the very high reactivity of the OH radical, allowing it to react with a wide range of molecules found in living cells. For several herbs, high correlation coefficients were obtained between the PDI value and the ability to neutralize free radicals, but this trend does not apply to the entire pool analyzed. For most herbs, it was possible to link their size with their antibacterial ability against both *E. coli* and *S. aureus*. However, high coefficients between the PDI value and the antibacterial capacity against E.coli were demonstrated only for blackberry (−0.964) and lemon balm (0.977). However, analogous correlation coefficients for *S. aureus* were determined for sage (0.706) and ribwort plantain (0.715). Conducting the synthesis at an elevated temperature significantly increased the correlations between the size of the nanoparticles and their homogeneity for yarrow (correlation coefficient 0.989), blackberry (0.999), nettle (0.906), lemon balm (0.835), and ribwort plantain (0.851). On the other hand, for raspberry and sage, low correlation coefficients were obtained between these values. An increased correlation was also observed between the ability to neutralize free radicals and the size of yarrow, sage, hops and ribwort plantain. At the same time, this correlation decreases for blackberry, nettle, and lemon balm. Interestingly, for most herbs, increasing the temperature of SeNP synthesis decreases the correlation between their ability to neutralize free radicals and their homogeneity.

### 2.3. The Differences in the Mechanism of Action Between the Chemically and Green Synthesized SeNPs

Selenium nanoparticles are considered a substitute for antibiotics, especially in the treatment of infections with antibiotic-resistant bacteria. This great potential of nanoparticles as antimicrobial agents can be explained by their ability to simultaneously act through multiple mechanisms, including the generation of radical oxygen species (ROS), interaction with cell barrier, or inhibition of the synthesis of proteins and DNA [45]. In such a situation, microbes are unable to develop resistance to these expressed mechanisms of action, contrary to conventional antibiotics. Our previous work indicates that green synthesized SeNPs exhibit antibacterial activity against *E. coli* [46]. One of the main mechanisms of their action is related to the formation of ROS in bacterial cells. Studied selenium nanoparticles showed strong inhibition of catalase, which is a crucial enzyme for bacterial cells that is involved in the removal of hydrogen peroxide. SeNPs also affected the reduction in the activity of superoxide dismutase, which is also involved in the removal of reactive oxygen species from cells. Green synthesized SeNPs were also shown to be involved in the cellular response to osmotic shock, confirming their pleiotropic mechanism of action in bacterial cells. Blinova et al. reported that chemically synthesized Se NPs-CTA leads to the degradation of proteins and polysaccharides and disruption of the microbial cell structure, allowing an enhanced penetration of Se NPs via damaged cytoplasmic membrane, which causes oxidative damage and changes in the intensity of reactive oxygen species [47]. Other authors have claimed that SeNPs inhibit the ability of bacteria to adhere to surfaces and form bacterial membranes, which can be used in the design of medical devices particularly susceptible to the formation of bacterial biofilms [48]. Han reported that the bactericidal activity of SeNPs is based on the degradation of microbial proteins [49]. It should be emphasized that in the case of the green synthesis of SeNPs, the resultant mechanism of their action is also influenced by the sample matrix. In this case, the residue of the plant extract is not separated from SeNPs. This extract determines the exceptionally strong stability of the nanoparticles obtained in this way. It should be assumed that research on this topic will be continued.

## 3. Materials and Methods

### 3.1. Obtaining the Plant Extract

The dried plant material used for nanoparticle synthesis was acquired from Kawon herb manufacturer, located in the Wielkopolska district. To prepare the extract, 5 g of ground dried plant material was weighed, and 50 mL of boiling water was added. Brewing was carried out for 30 min while stirring with the mixing intensity at 200 rpm. Before the synthesis, the extracts obtained were filtered through a filter paper.

### 3.2. Selenium Nanoparticle Synthesis

Nanoparticle synthesis was based on the reduction reaction of sodium selenite (Na_2_SO_3_) at a concentration of 0.1 mol L^−1^ with the plant extract. The reaction was carried out with variable ratios of reagents, so the exact procedure was as follows: 2.5 mL of sodium selenite solution was mixed with 15 mL of deionized water and placed on a magnetic stirrer (mixing intensity was set at 1000 rpm). Then, depending on the assumed ratio of reagents (1:1, 1:2, or 1:3), 2.5, 5.0, or 7.5 mL of herbal extract was added dropwise. The reaction mixture was stirred for 1 h. The synthesis reaction was carried out in two variants. The first assumed carrying out the entire synthesis at room temperature (25 °C), while the second included additional heating of the reaction mixture for half an hour (after one hour of stirring) at 70 °C for another hour. No additional stabilizers were included.

### 3.3. Characterization of Obtained Nanoparticles

The characterization of the obtained selenium nanoparticles was carried out using two methods: dynamic light scattering (DLS) and transmission electron microscopy (TEM, Thermo Fisher Scientific, Waltham, MA, USA). Dynamic light scattering measurements were performed using Mastersizer 2000 (Malvern, Panalutical, UK) instrumentation with a wet sample dispersion unit (Hydro 2000 MU, Malvern, Panalytical, UK). The range of nanoparticle size measurements started from particles larger than 0.01 μm (10 nm).

The zeta potential of obtained nanoparticles was measured by laser diffractometry using Zetasizer Nano ZS (Malvern, Panalytical, UK) under the following conditions: particle absorption coefficient of 0.01, a particle refractive index of 1.590, a water refractive index of 1.33, and temperature of 25 °C.

The morphology of the obtained SeNPs was studied using scanning electron microscopy (SEM) using field-emission SEM (Merlin Zeiss, Germany). Before measurements samples were plasma-sputtered with a few-nanometer-thick Au/Pd layer.

A more accurate measurement of the shape and size of the obtained nanoparticles was performed using transmission electron microscopy (TEM) with a TALOS F200 model (Thermo Fisher Scientific, Waltham, MA, USA) working at an accelerating voltage of 200 kV. Before the measurements, a drop of a post-reaction mixture containing synthesized SeNPs was placed on a copper grid and then air-dried before the examination. The results obtained were processed in the iTEMprogram, which is part of the apparatus software.

### 3.4. Antioxidant Activity Measurements

The antioxidant capacity of the obtained SeNPs was tested using four methods. Their capacity to neutralize OH and DPPH radicals was tested, as well as their reducing capacity using the CUPRAC method. Because the nanoparticles are so strongly stabilized by the post-reaction mixture, the total polyphenolic content was also determined using the Folin–Ciocalteu method.

Hydroxyl radical scavenging was performed based on the method described by Smirnoff and Cumbes [50]. The procedure was as follows: 1 mL of a selenium nanoparticles solution was mixed with 1 mL of iron sulfate (1.5 × 10^−3^ mol L^−1^), 0.7 mL of hydrogen peroxide (6 × 10^−3^ mol L^−1^), and 0.3 mL of sodium salicylate (2 × 10^−2^ mol L^−1^), and incubated at 37 °C for one hour. Then, the absorbance was measured at 562 nm. The obtained results were expressed as the percentage of OH radical scavenging.

The ability of the obtained SeNPs to neutralize free radicals was also tested using the DPPH radical scavenging method. In this assay, 0.1 mL of a nanoparticle solution was added to 2.4 mL of the methanolic radical solution (9 × 10^−5^ mol L^−1^). Then, the sample was kept for 30 min in the dark. Finally, the absorbance was measured at 518 nm. The obtained results are expressed as a Trolox equivalent (TRE) in μM.

The reducing capacity of the nanoparticle solution was evaluated using the CUPRAC method. The methodology was taken from Apak et al. [51]. In detail, 1 mL of copper chloride (1 × 10^−2^ mol L^−1^) was added to 1 mL of neocuproine methanolic solution (7.5 × 10^−3^ mol L^−1^) and then mixed with 1 mL of ammonium acetate buffer (1 M, pH 7). After that, 0.5 mL of herbal nanoparticle suspension and 0.6 mL of deionized water were added. The reaction mixture was incubated for 20 min in the water bath at 50 °C. Absorbance against the blank was measured at 450 nm. The obtained results are expressed as a Trolox equivalent (TRE) in μM.

Selenium nanoparticles obtained via green synthesis are so strongly stabilized by the post-reaction mixture that it is impossible to separate them from the solution as we reported earlier [20]. For this reason, the total polyphenol content in the nanoparticle suspension was also determined using the Folin–Ciocalteu method. Briefly, 1 mL of the SeNPs solution was mixed with 0.1 mL of FC reagent and 0.9 mL of water. After 5 min, 1 mL of 7% solution of Na_2_CO_3_ and 0.4 mL of water were added. After another 10 min, the absorbance was measured at 765 nm. The results obtained are expressed as a gallic acid (GA) equivalent.

Each sample in each assay was analyzed in triplicate.

Based on the results obtained, the antioxidant index (AOX) was calculated. For this purpose, the methodology described by Seeram et al. was used [52]. For all of the assays used, the appropriate index was calculated as a percentage of the average antioxidant capacities of a given sample compared to the highest (sample score/best score × 100). Finally, the AOX index was obtained for each sample as the sum of the individual indexes divided by four (the number of tests).

### 3.5. Antibacterial Activity Measurements

Escherichia coli MC1061 and Staphylococcus aureus ATCC 29213, obtained from the Institute of Microbiology (UW Department of Biology), were used for MIC (minimal inhibitory concentration) analyzes. Bacterial cultures were grown in LB medium or solid LB medium with 1.5% agar (BioMaxima, Poland). The MICs were determined using the classical serial microdilution method (Rohm and Haas RH-Europe RM-001-0608). The bacteria from 24 h LB agar cultures were suspended in saline to a 0.5 McFarland density, and test extracts were prepared in various concentrations (100%, 50%, 25%, 12%, 6.25%, 3.125%) in 96-well plates. E. coli or S. aureus suspensions were added, incubated at 37 °C with shaking for 24 h. MICs, defined as the lowest concentration inhibiting visible bacterial growth (no turbidity), were determined according to the CLSI guidelines [53]. Each test was repeated at least three times per bacterium.

### 3.6. Statistical Analysis and Data Presentation

The results of antioxidant activity measurements are expressed as the mean of three independent repetitions ± standard deviation. The significance of differences among means was set at a 5% probability level, determined using one-way ANOVA and Tukey’s test. All statistical tests were performed using E-stat software, available at http://beta.chem.uw.edu.pl/stat/ (accessed on 10 January 2025). All of the analyzed correlations were obtained using the Origin 8.5 program (Origin Lab Corporation, Northampton, USA). The uncertainty of the correlation coefficients was calculated based on the measured correlation and the amount of data. In each case the *p*-value was less than the significance level of α = 0.05.

## 4. Conclusions

Green synthesis is increasingly used to obtain selenium nanoparticles due to, among other reasons, the low toxicity of the reagents used. The obtained nanoparticles are characterized by high stability, which is visible in the high correlation results between the ratio of reagents used for synthesis and the size of the obtained SeNPs. This is also confirmed by the zeta potential values determined for the obtained SeNPs. Their values are also influenced by the concentration of the plant extract used for synthesis. All these observations confirm the potential of selenium nanoparticles in the biomedical field. However, the process itself is more difficult to control at the conducting stage due to the complexity of the chemical composition of the plant extract. Demonstrating the correlation between the antioxidant and antibacterial properties of the obtained nanoparticles and their physical properties is also more difficult because of the need to analyze the entire post-reaction mixture. Green synthesis offers well-stabilized nanoparticles without the need to use toxic stabilizers, but on the other hand, this makes it impossible to separate them from the mixture using conventional centrifugation. Determining correlations for multiple syntheses conducted using different herbs may distort certain relationships due to differences between the extracts, including differences in their polyphenol profile and content of other compounds. On the other hand, the correlations determined for individual herbs are sometimes contradictory to each other and inconsistent with those reported so far. However, it should be remembered that in many cases, these dependencies were determined for chemical syntheses. A better agreement of the obtained correlations with the theory was observed for the correlations determined for the SeNPs obtained at elevated temperature. However, further research is necessary to confirm this phenomenon. Moreover, further studies should focus on assessing the cytotoxicity of the selenium nanoparticles obtained. Some reports suggest that SeNPs exhibit concentration-dependent cytotoxicity, with low to moderate concentrations potentially stimulating cancer cell growth, while higher concentrations can induce cytotoxicity and apoptosis in cancer cells, often with selectivity over normal cells [54,55,56]. However, these reports concern only chemically synthesized SeNPs, not green synthesized SeNPs, with which some interactions with the sample matrix can have a significant influence on their mechanism of action.

## Figures and Tables

**Table 1:** Physical parameters and antioxidant and antibacterial activity of green synthesized SeNPs. Elevated temperatures are marked as H.

Herb		Physical Parameters			Antioxidant Properties					Antibacterial Activity	
	Reagent Ratio	Zeta Potential [mV]	Diameter [nm]	PDI	OH [%]	DPPH [mmolTr/L]	CUPRAC [mmolTr/L]	FC [mgGa/L]	AOX	*E. coli* [%]	*S. aureus* [%]
Yarrow (*Achillea* L.)	1:1	−22.5 ± 0.124	156	0.335	91.0 ± 3.60 ^a^	0.200 ± 0.004 ^a^	1.27 ± 0.03 ^a^	37.8 ± 0.831 ^a^	98.7 ± 2.57 ^a^	50	25
	1:2	−18.1 ± 0.368	161	0.054	98.2 ± 1.96 ^b^	0.642 ± 0.02 ^b^	1.16 ± 0.03 ^b^	40.0 ± 0.703 ^b^	96.1 ± 1.30 ^b^	50	25
	1:3	−17.7 ± 0.216	175	0.033	99.3 ± 3.44 ^c^	0.699 ± 0.02 ^c^	1.39 ± 0.01 ^c^	60.6 ± 0.912 ^c^	98.5 ± 2.71 ^a^	100	25
	1:1 H	−18.4 ± 0.653	110	0.232	89.2 ± 2.66 ^d^	0.290 ± 0.01 ^d^	0.82 ± 0.02 ^d^	35.1 ± 1.41 ^d^	93.3 ± 2.21 ^c^	50	6
	1:2 H	−15.7 ± 0.627	128	0.173	98.9 ± 2.95 ^b^	0.697 ± 0.03 ^e^	1.28 ± 0.02 ^a^	59.4 ± 1.01 ^e^	98.4 ± 1.08 ^a^	50	25
	1:3 H	−12.4 ± 0.417	146	0.036	90.6 ± 3.79 ^d^	0.701 ± 0.03 ^f^	1.40 ± 0.02 ^c^	60.1 ± 1.09 ^e^	97.7 ± 1.87 ^d^	100	25
Blackberry (*Rubus* L.)	1:1	−23.8 ± 1.02	96.8	0.150	99.0 ± 4.07 ^a^	0.748 ± 0.01 ^a^	1.37 ± 0.04 ^a^	32.4 ± 1.50 ^a^	98.5 ± 4.22 ^a^	25	12.5
	1:2	−20.1 ± 1.02	169	0.211	93.7 ± 3.95 ^b^	0.753 ± 0.03 ^b^	1.41 ± 0.03 ^b^	60.3 ± 2.85 ^b^	96.9 ± 4.09 ^b^	50	3
	1:3	−18.5 ± 1.35	175	0.309	84.7 ± 2.98 ^c^	0.748 ± 0.03 ^a^	1.40 ± 0.04 ^b^	104 ± 4.23 ^c^	99.4 ± 4.04 ^c^	25	6
	1:1 H	−23.4 ± 0.205	157	0.187	61.2 ± 2.95 ^d^	0.741 ± 0.02 ^c^	1.36 ± 0.04 ^c^	24.1 ± 1.05 ^c^	98.2 ± 3.96 ^a^	25	12.5
	1:2 H	−19.6 ± 0.317	171	0.268	89.9 ± 4.02 ^e^	0.751 ± 0.02 ^b^	1.42 ± 0.02 ^b^	52.2 ± 2.33 ^d^	95.7 ± 3.66 ^b^	50	3
	1:3 H	−18.7 ± 0.215	182	0.341	70.5 ± 3.32 ^f^	0.757 ± 0.02 ^d^	1.39 ± 0.03 ^b^	93.5 ± 4.21 ^e^	99.0 ± 4.32 ^c^	25	6
Sage (*Salvia officinalis* L.)	1:1	−13.1 ± 0.518	74.0	0.125	90.3 ± 3.98 ^a^	0.845 ± 0.04 ^a^	1.28 ± 0.05 ^a^	19.2 ± 0.831 ^a^	99.5 ± 3.87 ^a^	25	25
	1:2	−14.1 ± 0.464	164	0.190	99.2 ± 3.87 ^b^	0.740 ± 0.03 ^b^	1.39 ± 0.03 ^b^	46.9 ± 1.83 ^b^	98.7 ± 4.08 ^b^	12.5	12.5
	1:3	−14.5 ± 0.419	179	0.333	99.0 ± 3.76 ^c^	0.770 ± 0.03 ^c^	1.07 ± 0.04 ^c^	93.3 ± 4.08 ^c^	98.8 ± 4.20 ^b^	12.5	6
	1:1 H	−12.5 ± 0.490	93.1	0.212	91.7 ± 4.07 ^a^	0.725 ± 0.02 ^d^	1.36 ± 0.04 ^d^	18.7 ± 0.673 ^a^	97.5 ± 2.53 ^c^	50	25
	1:2 H	−12.4 ± 0.323	173	0.231	99.0 ± 3.32 ^c^	0.745 ± 0.02 ^b^	1.40 ± 0.05 ^e^	53.6 ± 2.31 ^d^	95.3 ± 3.77 ^d^	12.5	12.5
	1:3 H	−12.5 ± 0.402	182	0.397	99.0 ± 2.98 ^c^	0.750 ± 0.02 ^e^	1.40 ± 0.05 ^e^	76.6 ± 2.93 ^e^	97.6 ± 3.88 ^c^	12.5	6
Nettle (*Urtica* L.)	1:1	−29.4 ± 1.27	83.4	0.095	93.4 ± 2.80 ^a^	0.086 ± 0.004 ^a^	0.832 ± 0.02 ^a^	63.7 ± 2.13 ^a^	99.5 ± 2.18 ^a^	12.5	50
	1:2	−26.2 ± 1.02	155	0.193	91.5 ± 2.72 ^b^	0.193 ± 0.007 ^b^	1.21 ± 0.03 ^b^	47.8 ± 0.93 ^b^	97.5 ± 2.21 ^b^	12.5	12.5
	1:3	−24.3 ± 1.04	220	0.304	94.9 ± 3.80 ^c^	0.313 ± 0.009 ^c^	1.28 ± 0.04 ^c^	62.1 ± 1.74 ^a^	94.5 ± 1.91 ^c^	12.5	25
	1:1 H	−24.5 ± 0.680	140	0.100	95.2 ± 2.86 ^c^	0.113 ± 0.002 ^d^	0.884 ± 0.01 ^d^	51.6 ± 1.38 ^c^	93.4 ± 2.24 ^d^	12.5	25
	1:2 H	−22.5 ± 0.424	166	0.059	96.1 ± 3.81 ^d^	0.276 ± 0.01 ^e^	1.22 ± 0.03 ^b^	47.5 ± 0.87 ^b^	96.4 ± 3.61 ^e^	12.5	12.5
	1:3 H	−22.0 ± 0.697	140	0.090	91.6 ± 1.87 ^b^	0.187 ± 0.007 ^b^	1.39 ± 0.05 ^e^	76.5 ± 2.14 ^d^	97.6 ± 1.47 ^b^	25	12.5
Hop (*Humulus* L.)	1:1	−19.7 ± 0.805	84.9	0.109	99.0 ± 4.11 ^a^	0.289 ± 0.01 ^a^	0.957 ± 0.03 ^a^	11.2 ± 0.48 ^a^	97.7 ± 3.75 ^a^	50	12.5
	1:2	−19.2 ± 0.419	143	0.154	80.1 ± 3.35 ^b^	0.621 ± 0.02 ^b^	1.36 ± 0.03 ^b^	17.2 ± 0.73 ^b^	99.4 ± 4.00 ^b^	100	6
	1:3	−18.0 ± 0.411	170	0.322	77.8 ± 3.70 ^c^	0.749 ± 0.03 ^c^	1.41 ± 0.03 ^c^	17.3 ± 0.74 ^b^	99.0 ± 3.54 ^b^	NoI	6
	1:1 H	−18.2 ± 0.543	120	0.115	92.1 ± 3.25 ^d^	0.296 ± 0.02 ^d^	1.08 ± 0.02 ^d^	10.9 ± 0.45 ^c^	98.3 ± 3.98 ^c^	50	12.5
	1:2 H	−17.8 ± 0.410	165	0.195	77.6 ± 2.87 ^c^	0.666 ± 0.02 ^e^	1.39 ± 0.05 ^b^	17.8 ± 0.65 ^b^	96.6 ± 3.22 ^d^	100	6
	1:3 H	−17.2 ± 0.776	175	0.271	75.7 ± 2.92 ^e^	0.747 ± 0.02 ^c^	1.42 ± 0.04 ^c^	22.8 ± 1.03 ^d^	96.7 ± 2.91 ^d^	NoI	6
Lemon balm (*Melissa officinalis* L.)	1:1	−19.3 ± 1.07	79.0	0.103	98.0 ± 2.36 ^a^	0.810 ± 0.03 ^a^	0.840 ± 0.03 ^a^	98.5 ± 3.47 ^a^	99.6 ± 3.29 ^a^	100	50
	1:2	−17.0 ± 0.497	85.0	0.111	99.5 ± 3.95 ^b^	0.791 ± 0.02 ^b^	1.32 ± 0.04 ^b^	213 ± 8.51 ^b^	99.2 ± 4.08 ^a^	100	100
	1:3	−18.9 ± 0.826	113	0.172	83.1 ± 3.07 ^c^	0.790 ± 0.02 ^b^	1.43 ± 0.02 ^c^	101 ± 4.08 ^a^	99.1 ± 3.79 ^a^	NoI	100
	1:1 H	−15.5 ± 1.02	115	0.121	99.0 ± 3.33 ^b^	0.824 ± 0.03 ^c^	0.958 ± 0.04 ^e^	98.9 ± 2.36 ^a^	96.6 ± 2.41 ^b^	100	50
	1:2 H	−15.9 ± 0.632	158	0.210	99.0 ± 2.98 ^b^	0.824 ± 0.03 ^c^	1.41 ± 0.03 ^c^	216 ± 9.02 ^b^	98.2 ± 3.21 ^c^	100	100
	1:3 H	−16.6 ± 0.249	162	0.261	92.5 ± 4.04 ^d^	0.765 ± 0.02 ^d^	1.41 ± 0.03 ^c^	186 ± 3.33 ^c^	99.1 ± 3.84 ^b^	NoI	100
Ribwort plantain (*Plantago lanceolata* L.)	1:1	−13.8 ± 0.589	130	0.067	66.4 ± 2.12 ^a^	0.266 ± 0.006 ^a^	1.07 ± 0.03 ^a^	15.0 ± 0.411 ^a^	96.4 ± 2.02 ^a^	100	50
	1:2	−14.9 ± 1.18	162	0.054	52.7 ± 1.61 ^b^	0.766 ± 0.02 ^b^	1.44 ± 0.04 ^b^	27.9 ± 1.08 ^b^	97.5 ± 1.85 ^b^	100	100
	1:3	−14.6 ± 0.997	175	0.046	88.0 ± 3.50 ^c^	0.733 ± 0.02 ^c^	1.42 ± 0.03 ^b^	46.0 ± 1.32 ^c^	98.5 ± 2.05 ^c^	NoI	100
	1:1 H	−16.6 ± 1.23	163	0.040	64.4 ± 1.80 ^d^	0.292 ± 0.01 ^d^	1.12 ± 0.03 ^c^	16.8 ± 0.319 ^d^	93.7 ± 3.50 ^d^	100	50
	1:2 H	−13.4 ± 1.21	161	0.038	48.3 ± 1.42 ^e^	0.747 ± 0.02 ^e^	1.48 ± 0.02 ^d^	26.4 ± 0.933 ^b^	94.1 ± 2.44 ^e^	100	100
	1:3 H	−11.8 ± 1.25	149	0.035	84.0 ± 2.52 ^f^	0.719 ± 0.01 ^f^	1.40 ± 0.04 ^b^	44.7 ± 0.893 ^c^	96.8 ± 1.97 ^a^	NoI	100
Raspberry (*Rubus idaeus* L.)	1:1	−19.9 ± 0.818	74.0	0.132	89.2 ± 3.23 ^a^	0.392 ± 0.01 ^a^	1.39 ± 0.06 ^a^	15.4 ± 0.63 ^a^	98.3 ± 4.15 ^a^	25	25
	1:2	−19.0 ± 1.23	109	0.184	98.2 ± 4.32 ^b^	0.854 ± 0.04 ^b^	1.37 ± 0.04 ^b^	36.9 ± 1.60 ^b^	94.9 ± 3.54 ^b^	12.5	12.5
	1:3	−20.9 ± 0.741	124	0.141	99.0 ± 4.33 ^b^	0.798 ± 0.02 ^c^	1.06 ± 0.02 ^c^	72.0 ± 2.37 ^c^	98.5 ± 3.47 ^a^	12.5	12.5
	1:1 H	−16.6 ± 0.822	84.1	0.175	86.9 ± 4.20 ^c^	0.492 ± 0.02 ^d^	1.34 ± 0.03 ^d^	14.4 ± 0.51 ^d^	96.0 ± 3.89 ^c^	50	50
	1:2 H	−15.3 ± 0.713	167	0.190	81.6 ± 3.70 ^d^	0.773 ± 0.02 ^e^	1.37 ± 0.03 ^e^	37.6 ± 1.73 ^b^	99.3 ± 4.24 ^d^	12.5	25
	1:3 H	−14.7 ± 0.616	181	0.263	30.7 ± 0.992 ^e^	0.746 ± 0.01 ^f^	1.38 ± 0.04 ^e^	72.1 ± 2.75 ^c^	99.0 ± 4.18 ^d^	12.5	6

Results are expressed as the mean ± SD of three independent repetitions. Different letters in each section indicate a difference at a significance level of *p* = 0.05.

**Table 2:** Correlation coefficients between the physical parameters and antioxidant and antibacterial properties of SeNPs obtained under A room temperature and B elevated temperature.

	Correlation Coefficients (R^2^)										
A	Size	PDI	Ratio	ζ-Potential	FC	CUPRAC	DPPH	OH	AOX	MIC *E. coli*	MIC *S. aureus*
Size		0.148	0.937	−0.021	−0.043	0.103	−0.045	−0.024	0.026	−0.060	−0.041
PDI	0.148		0.681	−0.113	−0.036	−0.019	−0.042	−0.033	−0.045	0.173	0.203
Ratio	0.681	0.681		0.936	0.693	0.522	−0.850	0.659	−0.998	−0.850	0.435
ζ-potential	−0.021	−0.113	0.936		−0.045	0.057	0.303	−0.303	−0.041	0.039	0.015
FC	−0.043	−0.036	0.693	−0.045		−0.042	0.047	0.319	0.031	−0.026	0.120
CUPRAC	0.103	−0.019	0.522	0.057	−0.042		0.138	0.033	0.039	−0.030	−0.035
DPPH	−0.045	−0.042	−0.850	0.303	0.047	0.138		−0.085	−0.039	−0.043	−0.020
OH	−0.024	−0.033	0.659	−0.303	0.319	0.033	−0.085		0.045	0.038	0.107
AOX	0.026	−0.045	−0.998	−0.041	0.031	0.039	−0.039	0.045		−0.040	−0.020
MIC *E. coli*	−0.060	0.173	−0.850	0.030	−0.026	−0.030	−0.043	0.038	−0.040		0.011
MIC *S. aureus*	−0.041	0.203	0.435	0.015	0.120	−0.035	−0.020	0.107	−0.020	0.011	
	Correlation Coefficients (R^2^)										
B	Size	PDI	Ratio	ζ-potential	FC	CUPRAC	DPPH	OH	AOX	MIC *E. coli*	MIC *S. aureus*
Size		0.052	0.914	−0.045	0.008	0.181	0.056	0.069	−0.009	0.027	0.027
PDI	0.052		0.880	−0.038	0.033	0.026	0.178	−0.044	0.101	0.078	−0.044
Ratio	0.914	0.880		0.784	0.716	0.031	0.964	0.362	−0.996	0.850	0.435
ζ-potential	−0.045	−0.038	0.784		−0.043	0.109	0.333	−0.040	−0.021	−0.006	0.008
FC	0.008	0.033	0.716	−0.043		−0.035	0.048	0.026	0.103	−0.045	0.179
CUPRAC	0.181	0.026	0.031	0.109	−0.035		0.361	0.023	0.217	−0.030	−0.010
DPPH	0.056	0.178	0.964	0.333	0.048	0.361		−0.018	0.217	−0.041	0.013
OH	0.069	−0.044	0.362	−0.040	0.026	0.023	−0.018		−0.045	−0.044	−0.045
AOX	−0.009	0.101	−0.996	−0.021	0.103	0.217	0.217	−0.045		0.027	−0.043
MIC *E. coli*	0.027	0.078	0.850	−0.006	−0.045	−0.030	−0.041	−0.044	0.027		0.016
MIC *S. aureus*	0.027	−0.044	0.435	0.008	0.179	−0.010	0.013	−0.045	−0.043	0.016	

**Table 3:** Correlation coefficients between the physical parameters and antioxidant and antibacterial properties of SeNPs obtained under A room temperature and B elevated temperatures designated for individual herbs.

Herb		Correlation Coefficients (R^2^)										
A		Size	PDI	Ratio	ζ-Potential	FC	CUPRAC	DPPH	OH	AOX	MIC *E. coli*	MIC *S. aureus*
Yarrow (*Achillea* L.)	Size		−0.746	−0.853	0.124	0.943	0.069	0.197	0.232	−0.923	0.871	-
	PDI	−0.746		0.932	0.999	−0.222	−0.746	0.996	0.993	−0.488	−0.388	-
	Ratio	−0.853	0.932		0.936	0.146	−0.851	0.960	0.969	−0.770	−0.031	-
	ζ-potential	0.124	0.999	0.936		−0.212	−0.981	0.997	0.993	−0.497	−0.379	0.990
	FC	0.943	−0.222	0.146	−0.212		0.356	−0.139	−0.104	−0.742	0.985	-
	CUPRAC	0.069	−0.983	−0.851	−0.981	0.356		−0.964	−0.954		0.550	-
	DPPH	0.197	0.996	0.960	0.997	−0.139	−0.964		0.999	0.321	0.507	-
	OH	0.232	0.993	0.969	0.993	−1.04	−0.954	0.999		0.321	−0.275	-
	AOX	−0.923	−0.488	−0.770	−0.497	−0.742	0.321	0.321	−0.589		−0.615	-
	MIC *E. coli*	0.871	−0.388	−0.031	−0.379	0.985	0.550	−0.309	−0.275	−0.615		-
	MIC *S. aureus*	0.864	-	-	0.990	-	-	-	-	-	-	
		Size	PDI	Ratio	ζ-potential	FC	CUPRAC	DPPH	OH	AOX	MIC *E. coli*	MIC *S. aureus*
Blackberry (*Rubus* L.)	Size		0.384	0.937	0.896	0.399	0.786	−0.405	0.356	−0.983	−0.614	−0.721
	PDI	0.384		0.681	0.753	0.999	−0.268	−0.964	0.999	−0.545	−0.964	−0.383
	Ratio	0.937	0.681		0.994	0.693	0.522	−0.850	0.659	−0.998	0.850	0.435
	ζ-potential	0.896	0.753	0.994		0.763	0.430	−0.900	0.734	−0.962	−0.900	0.339
	FC	0.399	0.999	0.693	0.763		−0.253	−0.968	0.999	−0.559	−0.968	−0.348
	CUPRAC	0.786	−0.268	0.522	0.430	−0.253		0.005	−0.297	−0.661	0.005	0.995
	DPPH	−0.405	−0.964	−0.850	−0.900	−0.968	0.005		0.709	−0.660	−0.472	−0.926
	OH	0.356	0.999	0.659	0.734	0.999	−0.297	0.709		−0.521	−0.956	−0.390
	AOX	−0.983	−0.545	−0.998	−0.962	−0.559	−0.661	−0.660	−0.521		0.747	−0.582
	MIC *E. coli*	−0.614	−0.964	0.850	−0.900	−0.125	0.500	−0.472	−0.956	0.747		0.104
	MIC *S. aureus*	−0.721	−0.383	0.435	0.339	−0.968	0.913	−0.926	−0.390	−0.582	0.104	
		Size	PDI	Ratio	ζ-potential	FC	CUPRAC	DPPH	OH	AOX	MIC *E. coli*	MIC *S. aureus*
Sage (*Salvia officinalis* L.)	Size		0.354	0.974	0.701	0.478	−0.790	0.677	0.954	−0.880	0.965	0.912
	PDI	0.354		0.556	−0.417	0.991	0.293	0.448	0.057	−0.133	0.097	0.706
	Ratio	0.974	0.556		0.522	0.685	−0.631	0.493	0.880	0.750	0.881	0.981
	ζ-potential	0.701	−0.417	0.522		−0.289	−0.991	0.999	0.882	0.956	0.863	0.348
	FC	0.478	0.991	0.685	−0.289		0.159	−0.322	0.194	0.004	0.232	0.796
	CUPRAC	−0.790	0.293	−0.631	−0.991	0.159		−0.985	−0.937	−0.986	−0.923	−0.471
	DPPH	0.677	0.448	0.493	0.999	−0.322	−0.985		0.866	−0.986	0.846	0.317
	OH	0.954	0.057	0.880	0.882	0.194	−0.937	0.866		0.982	0.999	0.748
	AOX	−0.880	−0.133	0.750	0.956	0.004	−0.986	−0.986	0.982		0.974	0.608
	MIC *E. coli*	0.965	0.097	0.881	0.863	0.232	−0.923	0.846	0.999	0.974		0.773
	MIC *S. aureus*	0.912	0.706	0.981	0.348	0.796	−0.471	0.317	0.748	0.608	0.773	
		Size	PDI	Ratio	ζ-potential	FC	CUPRAC	DPPH	OH	AOX	MIC *E. coli*	MIC *S. aureus*
Nettle (*Urtica* L.)	Size		0.906	0.875	0.972	−0.972	0.761	0.992	−0.655	0.959	−0.998	−0.879
	PDI	0.906		0.810	0.934	−0.993	0.672	0.999	−0.554	0.987	-	−0.213
	Ratio	0.875	0.810		0.965	−0.740	0.979	0.815	−0.938	0.708	-	0.339
	ζ-potential	0.972	0.934	0.965		−0.889	0.892	0.937	−0.813	0.867	0.728	0.148
	FC	−0.972	−0.993	−0.740	−0.889		−0.586	−0.992	0.458	−0.999	-	0.321
	CUPRAC	0.761	0.672	0.979	0.892	−0.586		0.678	−0.988	0.547	-	0.579
	DPPH	0.992	0.999	0.815	0.937	−0.992	0.678		−0.560	0.548	-	0.706
	OH	−0.655	−0.554	−0.938	−0.813	0.458	−0.988	−0.560		−0.417	-	−0.694
	AOX	0.959	0.987	0.708	0.867	−0.999	0.547	0.548	−0.417		-	−0.304
	MIC *E. coli*	−0.998	-	-	0.728	-	-	-	-	-		0.785
	MIC *S. aureus*	−0.879	−0.213	0.399	0.148	0.321	0.579	0.706	−0.694	−0.364	0.785	
		Size	PDI	Ratio	ζ-potential	FC	CUPRAC	DPPH	OH	AOX	MIC *E. coli*	MIC *S. aureus*
Hop (*Humulus* L.)	Size		0.489	0.353	0.703	0.823	0.909	0.996	0.907	−0.907	0.807	0.807
	PDI	0.489		0.384	0.964	−0.090	0.081	0.413	0.078	−0.530	0.120	−0.119
	Ratio	0.353	0.384		−0.597	0.894	0.957	0.999	0.956	0.584	−0.879	0.880
	ζ-potential	0.703	0.964	−0.597		0.175	0.342	0.640	0.339	−0.301	−0.147	0.147
	FC	0.823	−0.090	0.894	0.175		0.985	0.869	0.986	0.885	−0.999	0.999
	CUPRAC	0.909	0.081	0.957	0.342	0.985		0.941	−0.999	0.793	−0.979	0.980
	DPPH	0.996	0.413	0.999	0.640	0.869	0.941		0.940	0.793	−0.854	0.854
	OH	0.907	0.078	0.956	0.339	0.986	−0.999	0.940		0.794	−0.980	0.980
	AOX	−0.908	−0.530	0.584	−0.301	0.885	0.793	0.793	0.794		−0.898	0.898
	MIC *E. coli*	0.807	0.120	−0.879	−0.147	−0.999	−0.979	−0.854	−0.980	−0.898		−1
	MIC *S. aureus*	0.807	−0.119	0.880	0.147	0.999	0.980	0.854	0.980	0.898	−1	
		Size	PDI	Ratio	ζ-potential	FC	CUPRAC	DPPH	OH	AOX	MIC *E. coli*	MIC *S. aureus*
Lemon balm (*Melissa officinalis* L.)	Size		0.993	−0.805	−0.901	−0.778	0.160	−0.102	0.878	0.187	0.945	−0.190
	PDI	0.993		−0.768	−0.842	−0.698	0.041	0.221	0.924	0.069	0.977	−0.306
	Ratio	−0.805	0.180		0.682	−0.830	0.990	0.919	−0.204	0.994	−0.030	0.881
	ζ-potential	−0.901	−0.842	0.682		0.974	−0.572	−0.990	−0.584	−0.595	−0.710	−0.255
	FC	−0.778	−0.698	−0.830	0.974		−0.744	−0.544	−0.384	−0.762	−0.531	−0.531
	CUPRAC	0.160	0.041	0.990	−0.572	−0.744		−0.991	−0.330	0.999	−0.170	0.938
	DPPH	−0.102	0.221	0.919	−0.990	−0.544	−0.991		−0.585	−0.996	−0.421	0.996
	OH	0.878	0.924	−0.204	−0.584	−0.384	−0.330	−0.585		−0.304	0.986	0.773
	AOX	0.187	0.069	0.994	−0.595	−0.762	0.999	−0.996	−0.304		−0.143	0.928
	MIC *E. coli*	0.945	0.977	−0.030	−0.710	−0.531	−0.170	−0.421	0.986	−0.143		−0.500
	MIC *S. aureus*	−0.190	−0.306	0.881	−0.255	−0.467	0.938	0.996	0.773	0.928	−0.500	
		**Size**	**PDI**	**Ratio**	**ζ-potential**	**FC**	**CUPRAC**	**DPPH**	**OH**	**AOX**	**MIC *E. coli***	**MIC *S. aureus***
Ribwort Plantain (*Plantago lanceolata* L.)	Size		−0.986	0.999	0.292	0.711	0.844	0.837	−0.752	0.862	−0.062	0.895
	PDI	−0.986		0.961	0.091	0.893	0.638	0.628	−0.514	0.976	0.247	0.715
	Ratio	0.999	0.961		0.364	0.734	0.827	0.818	−0.732	0.878	−0.031	0.881
	ζ-potential	0.292	0.091	0.364		−0.365	0.825	0.833	−0.901	−0.127	−0.942	0.762
	FC	0.711	0.893	0.734	−0.365		0.224	0.211	−0.073	0.969	0.656	0.325
	CUPRAC	0.844	0.638	0.827	0.825	0.224		0.999	−0.988	0.457	−0.587	0.994
	DPPH	0.837	0.628	0.818	0.833	0.211	0.999		0.999	0.457	−0.598	0.993
	OH	−0.752	−0.514	−0.732	−0.901	−0.073	−0.988	0.999		−0.316	0.704	−0.967
	AOX	0.862	0.976	0.878	−0.127	0.969	0.457	0.457	−0.316		0.452	0.547
	MIC *E. coli*	−0.062	0.247	−0.031	−0.942	0.656	−0.587	−0.598	0.704	0.452		−0.500
	MIC *S. aureus*	0.895	0.715	0.881	0.762	0.325	0.994	0.993	−0.967	0.547	−0.500	
		Size	PDI	Ratio	ζ-potential	FC	CUPRAC	DPPH	OH	AOX	MIC *E. coli*	MIC *S. aureus*
Raspberry (*Rubus idaeus* L.)	Size		−0.711	−0.998	−0.779	0.745	0.162	0.685	0.902	−0.937	0.829	0.829
	PDI	−0.711		−0.637	0.114	−0.998	0.041	0.221	−0.339	0.911	−0.634	−0.197
	Ratio	−0.998	−0.637		−0.836	0.675	0.078	0.776	−0.985	−0.898	0.881	0.887
	ζ-potential	−0.779	0.114	−0.836		−0.164	0.492	−0.973	0.513	0.513	−0.996	−0.996
	FC	0.745	−0.998	0.675	−0.164		0.778	0.025	0.584	−0.931	0.245	0.245
	CUPRAC	0.162	0.041	0.078	0.492	0.778		−0.608	−0.280	−0.495	−0.417	−0.418
	DPPH	0.685	0.221	0.776	−0.973	0.025	−0.608		0.932	0.495	0.975	0.975
	OH	0.902	−0.339	−0.985	0.513	0.584	−0.280	0.932		−0.695	0.989	0.989
	AOX	−0.937	0.911	−0.898	0.513	−0.931	−0.495	0.495	−0.695		−0.583	−0.583
	MIC *E. coli*	0.829	−0.634	0.881	−0.996	0.245	−0.417	0.975	0.989	−0.583		1
	MIC *S. aureus*	0.829	−0.197	0.887	−0.996	0.245	−0.418	0.975	0.989	−0.583	1	
Herb		Correlation Coefficients (R^2^)										
B		Size	PDI	Ratio	ζ-potential	FC	CUPRAC	DPPH	OH	AOX	MIC *E. coli*	MIC *S. aureus*
Yarrow (*Achillea* L.)	Size		0.989	0.843	0.993	0.537	0.786	0.509	−0.966	0.999	0.506	0.864
	PDI	0.989		0.761	0.943	0.408	0.687	0.378	−0.993	0.117	0.625	0.363
	Ratio	0.843	0.761		0.784	0.903	0.994	0.880	−0.680	0.734	−0.031	0.881
	ζ-potential	0.993	0.943	0.784		0.442	0.714	0.413	−0.988	0.154	0.596	0.397
	FC	0.537	0.408	0.903	0.442		0.943	0.999	−0.300	0.954	−0.456	0.988
	CUPRAC	0.786	0.687	0.994	0.714	0.943		0.932	−0.600	0.801	−0.136	0.926
	DPPH	0.509	0.378	0.880	0.413	0.999	0.932		−0.269	0.801	−0.485	0.999
	OH	−0.966	−0.993	−0.680	−0.988	−0.300	−0.600	−0.269		−0.002	−0.711	−0.253
	AOX	0.999	0.117	0.734	0.154	0.954	0.801	0.801	−0.002		−0.701	0.967
	MIC *E. coli*	0.506	0.625	−0.031	0.596	−0.456	−0.136	−0.485	−0.711	−0.701		−0.500
	MIC *S. aureus*	0.864	0.363	0.881	0.397	0.988	0.926	0.999	−0.253	0.967	−0.500	
		Size	PDI	Ratio	ζ-potential	FC	CUPRAC	DPPH	OH	AOX	MIC *E. coli*	MIC *S. aureus*
Blackberry (*Rubus* L.)	Size		0.999	0.825	0.849	0.937	−0.375	0.989	0.707	−0.946	−0.990	0.034
	PDI	0.999		0.880	0.805	0.961	−0.447	0.974	−0.760	−0.917	−0.998	−0.044
	Ratio	0.825	0.880		0.990	0.716	0.031	0.964	−0.387	−0.996	0.850	0.435
	ζ-potential	0.849	0.805	0.990		0.610	0.171	0.918	−0.227	−0.974	−0.769	0.557
	FC	0.937	0.961	0.716	0.610		−0.798	0.875	−0.970	−0.645	−0.921	−0.484
	CUPRAC	−0.375	−0.447	0.031	0.171	−0.798		−0.235	0.921	0.054	0.500	0.913
	DPPH	0.989	0.974	0.964	0.918	0.875	−0.235		−0.595	0.054	−0.960	0.181
	OH	0.707	−0.760	−0.387	−0.227	−0.970	0.921	−0.595		0.440	0.798	0.682
	AOX	−0.946	−0.917	−0.996	−0.974	−0.645	0.054	0.054	0.440		0.892	−0.360
	MIC *E. coli*	−0.990	−0.998	0.850	−0.769	−0.921	0.500	−0.960	0.798	0.892		0.104
	MIC *S. aureus*	0.034	−0.044	0.435	0.557	−0.484	0.913	0.181	0.682	−0.360	0.104	
		Size	PDI	Ratio	ζ-potential	FC	CUPRAC	DPPH	OH	AOX	MIC *E. coli*	MIC *S. aureus*
Sage (*Salvia officinalis* L.)	Size		−0.152	0.953	−0.983	0.810	0.983	0.980	0.981	−0.880	0.983	0.876
	PDI	−0.152		0.155	0.390	0.456	−0.331	0.436	−0.330	−0.592	−0.330	0.342
	Ratio	0.953	0.155		−0.880	0.950	−0.880	0.994	0.857	−0.883	0.881	0.981
	ζ-potential	−0.983	0.390	−0.880		−0.689	−1	−0.928	−1	0.565	−1	−0.773
	FC	0.810	0.456	0.950	−0.689		0.689	0.908	0.689	−0.987	0.687	0.992
	CUPRAC	0.983	−0.331	−0.880	−1	0.689		0.928	1	−0.565	−1	0.773
	DPPH	0.980	0.436	0.994	−0.928	0.908	0.928		0.929	−0.565	0.928	0.953
	OH	0.981	−0.330	0.857	−1	0.689	1	0.929		−0.565	1	0.748
	AOX	−0.880	−0.592	−0.883	0.565	−0.987	−0.565	−0.565	−0.565		−0.565	−0.960
	MIC *E. coli*	0.983	−0.330	0.881	−1	0.687	−1	0.928	1	−0.565		0.773
	MIC *S. aureus*	0.876	0.342	0.981	−0.773	0.992	0.773	0.953	0.748	−0.960	0.773	
		Size	PDI	Ratio	ζ-potential	FC	CUPRAC	DPPH	OH	AOX	MIC *E. coli*	MIC *S. aureus*
Nettle (*Urtica* L.)	Size		0.906	−0.829	−0.788	0.585	−0.919	0.898	−0.176	−0.868	−0.528	0.471
	PDI	0.906		−0.518	−0.423	−0.669	−0.664	0.892	−0.577	−0.575	−0.839	−0.051
	Ratio	−0.829	−0.518		0.994	−0.288	0.983	−0.075	−0.399	0.997	−0.031	0.881
	ζ-potential	−0.788	−0.423	0.994		−0.968	0.958	0.032	−0.495	0.984	−0.138	0.927
	FC	0.585	−0.669	−0.288	−0.968		−0.857	−0.278	0.695	−0.910	0.378	0.321
	CUPRAC	−0.919	−0.664	0.983	0.958	−0.857		−0.255	−0.226	0.993	0.151	0.780
	DPPH	0.898	0.892	−0.075	0.032	−0.278	−0.255		−0.884	0.983	−0.994	0.405
	OH	−0.176	−0.577	−0.399	−0.495	0.695	−0.226	−0.884		−0.417	0.928	0.785
	AOX	−0.868	−0.575	0.997	0.984	−0.910	0.993	0.983	−0.417		0.038	0.846
	MIC *E. coli*	−0.528	−0.839	−0.031	−0.138	0.378	0.151	−0.994	0.928	0.038		−0.500
	MIC *S. aureus*	0.471	−0.051	0.881	0.927	0.321	0.780	0.405	0.785	0.846	−0.500	
		Size	PDI	Ratio	ζ-potential	FC	CUPRAC	DPPH	OH	AOX	MIC *E. coli*	MIC *S. aureus*
Hop (*Humulus* L.)	Size		0.781	0.951	0.517	0.867	0.983	0.999	0.999	0.901	−0.941	−0.941
	PDI	0.781		0.865	0.938	0.988	0.654	0.778	0.692	0.433	−0.525	0.525
	Ratio	0.951	0.865		0.639	0.932	0.944	0.988	0.961	0.826	−0.880	0.880
	ζ-potential	0.517	0.938	0.639		0.876	0.353	0.514	0.402	0.096	−0.200	0.200
	FC	0.867	0.988	0.932	0.876		0.762	0.865	0.795	0.566	−0.649	0.649
	CUPRAC	0.983	0.654	0.944	0.353	0.762		0.984	0.998	0.965	−0.987	0.987
	DPPH	0.999	0.778	0.988	0.514	0.865	0.984		0.992	0.965	−0.943	0.943
	OH	0.999	0.692	0.961	0.402	0.795	0.998	0.992		0.794	−0.977	0.977
	AOX	0.901	0.433	0.826	0.096	0.566	0.965	0.965	0.794		−0.994	0.994
	MIC *E. coli*	−0.941	−0.525	−0.880	−0.200	−0.649	−0.987	−0.943	−0.977	−0.994		−1
	MIC *S. aureus*	−0.941	0.525	0.880	0.200	0.649	0.987	0.943	0.977	0.994	−1	
		Size	PDI	Ratio	ζ-potential	FC	CUPRAC	DPPH	OH	AOX	MIC *E. coli*	MIC *S. aureus*
Lemon balm (*Melissa officinalis* L.)	Size		0.835	0.942	0.357	0.795	0.988	−0.361	−0.361	0.840	−0.361	0.988
	PDI	0.835		0.970	0.812	0.329	0.741	0.211	0.211	0.999	0.211	0.741
	Ratio	0.942	0.970		0.647	0.547	0.880	−0.031	−0.031	0.972	−0.030	0.881
	ζ-potential	0.357	0.812	0.647		−0.283	0.210	0.742	0.742	0.806	0.742	0.210
	FC	0.795	0.329	0.547	−0.283		0.878	−0.859	−0.853	0.339	−0.853	0.878
	CUPRAC	0.988	0.741	0.880	0.210	0.878		−0.500	−0.500	0.747	−0.500	1
	DPPH	−0.361	0.211	−0.031	0.742	−0.859	−0.500		1	0.747	1	−0.500
	OH	−0.361	0.211	−0.031	0.742	−0.853	−0.500	1		0.202	1	0.630
	AOX	0.840	0.999	0.972	0.806	0.339	0.747	0.747	0.202		0.202	0.747
	MIC *E. coli*	−0.361	0.211	−0.030	0.742	−0.853	−0.500	1	1	0.202		−0.500
	MIC *S. aureus*	0.988	0.741	0.881	0.210	0.878	1	−0.500	0.630	0.747	−0.500	
		Size	PDI	Ratio	ζ-potential	FC	CUPRAC	DPPH	OH	AOX	MIC *E. coli*	MIC *S. aureus*
Ribwort Plantain (*Plantago lanceolata* L.)	Size		0.851	0.225	0.388	0.911	−0.630	−0.360	0.363	0.999	0.965	−0.255
	PDI	0.851		0.708	0.814	0.992	−0.128	0.183	−0.180	0.836	0.684	0.289
	Ratio	0.225	0.708		0.986	0.613	0.606	0.824	−0.821	0.206	−0.031	0.881
	ζ-potential	0.388	0.814	0.986		0.734	0.472	0.720	−0.717	0.383	0.134	0.791
	FC	0.911	0.992	0.613	0.734		−0.251	0.057	−0.054	0.969	0.771	0.167
	CUPRAC	−0.630	−0.128	0.606	0.472	−0.251		0.952	−0.952	−0.650	−0.811	0.912
	DPPH	−0.360	0.183	0.824	0.720	0.057	0.952		−0.999	−0.650	0.591	0.993
	OH	0.363	−0.180	−0.821	−0.717	−0.054	−0.952	−0.999		0.388	0.594	−0.993
	AOX	0.999	0.836	0.206	0.383	0.969	−0.650	−0.650	0.388		0.972	−0.282
	MIC *E. coli*	0.965	0.684	−0.031	0.134	0.771	−0.811	0.591	0.594	0.972		0.011
	MIC *S. aureus*	−0.255	0.289	0.881	0.791	0.167	0.912	0.993	−0.993	−0.282	0.011	
		Size	PDI	Ratio	ζ-potential	FC	CUPRAC	DPPH	OH	AOX	MIC *E. coli*	MIC *S. aureus*
Raspberry (*Rubus idaeus* L.)	Size		0.064	0.974	0.989	0.536	0.873	0.904	0.080	−0.937	0.964	0.813
	PDI	0.064		0.285	0.352	0.876	0.544	−0.369	0.989	−0.360	−0.202	0.634
	Ratio	0.974	0.285		−0.997	0.711	0.960	0.784	0.140	−0.898	0.881	0.922
	ζ-potential	0.989	0.352	−0.997		0.759	0.977	0.740	−0.209	0.746	0.845	0.947
	FC	0.536	0.876	0.711	0.759		0.881	0.124	0.794	0.133	0.294	0.928
	CUPRAC	0.873	0.544	0.960	0.977	0.881		0.579	0.413	0.587	0.712	0.994
	DPPH	0.904	−0.369	0.784	0.740	0.124	0.579		−0.503	0.587	0.985	0.484
	OH	0.080	0.989	0.140	−0.209	0.794	0.413	−0.503		−0.695	−0.345	0.512
	AOX	−0.937	−0.360	−0.898	0.746	0.133	0.587	0.587	−0.695		0.986	0.493
	MIC *E. coli*	0.964	−0.202	0.881	0.845	0.294	0.712	0.985	−0.345	0.986		1
	MIC *S. aureus*	0.813	0.634	0.922	0.947	0.928	0.994	0.484	0.512	0.493	1	

## Data Availability

The data presented in this study are available on request from the corresponding author.

## Supplementary Materials

The following supporting information can be downloaded at https://www.mdpi.com/article/10.3390/molecules30132865/s1: Table S1. The polyphenolic content in the herbal infusions used in the synthesis of SeNP.
