# Supplementary material for: Is There Any Correlation Between Green Synthesis Parameters and the Properties of Obtained Selenium Nanoparticles?
Source: Molecules. 2025 Jul 5;30(13):2865. doi: 10.3390/molecules30132865 (PMC12250725; doi:10.3390/molecules30132865)
Supplement: Supplementary file 1 [file molecules-30-02865-s001.zip › molecules-3710283-supplementary.pdf]

Supplementary material

Is there any correlation between green synthesis parameters and the properties of obtained selenium nanoparticles?

Aleksandra Sentkowska, Julia Folcik, Jakub Szmytke,Anna Grudniak

Table S1. The polyphenolic content in the herbal infusions used in the synthesis of SeNP.

	**Plant material used for SeNPs synthesis**							
	**Blackberry**	**Hop**	**Lemon balm**	**Raspberry**	**Sage**	**Yarrow**	**Nettle**	**Ribsworth plantatin**
Flavonoids*								
Kaempferol	<LOD	0.705 ± 0.023^{a}	0.507 ± 0.020^{b}	<LOD	0.748± 0.030^{a}	0.732± 0.014^{a}	<LOD	<LOD
Epicatechin	9.62 ± 0.324^{a}	7.54 ± 0.295^{b}	3.00 ± 0.101^{c}	17.7 ± 0.730^{d}	5.08 ± 0.220^{e}	3.49 ± 0.133^{f}	2.54 ± 0.102^{g}	2.72 ± 0.133^{g}
Catechin	3.91 ± 0.183^{a}	1.22 ± 0.045^{b}	0.11 ± 0.003^{c}	2.02 ± 0.092^{d}	0.10 ± 0.002^{c}	<LOD	0.797 ± 0.032^{e}	<LOD
EGCG	0.197 ± 0.007^{a}	<LOD	<LOD	<LOD	0.180 ± 0.040^{b}	<LOD	<LOD	<LOD
Quercetin	0.064 ± 0.003^{a}	0.608 ± 0.025^{b}	0.078 ± 0.002^{c}	0.077 ± 0.003^{c}	0.052 ± 0.002^{d}	0.074 ±0.002^{c}	0.054 ± 0.003^{d}	<LOD
Naringenin	<LOD	2.47 ± 0.100^{a}	<LOD	0.237 ± 0.011^{b}	0.312 ± 0.014^{c}	<LOD	<LOD	0.373 ± 0.016^{c}
Hesperetin	<LOD	8.18 ± 0.305^{a}	<LOD	<LOD	<LOD	<LOD	<LOD	<LOD
Myricetin	<LOD	<LOD	0.087 ± 0.003^{a}	<LOD	0.161 ± 0.006^{b}	<LOD	0.055 ± 0.002^{c}	<LOD
Apigenin	0.09 ± 0.003^{a}	<LOD	0.138 ± 0.004^{b}	<LOD	1.05 ± 0.041^{c}	1.14 ± 0.025^{c}	<LOD	0.425 ±0.011^{d}
Naringin	<LOD	<LOD	<LOD	<LOD	0.717± 0.024^{a}	<LOD	<LOD	0.205 ± 0.010^{b}
Luteolin	0.15 ± 0.006^{a}	<LOD	<LOD	0.06 ± 0.002^{b}	2.01 ± 0.093^{c}	<LOD	<LOD	0.056 ± 0.002^{b}
Rutin	1.45 ± 0.064^{a}	<LOD	<LOD	2.49 ± 0.102^{b}	0.347 ± 0.012^{c}	3.42 ± 0.121^{d}	<LOD	<LOD
Polyphenolic acid*								
Chlorogenic acid	7.58 ± 0.325^{a}	2.93 ± 0.110^{b}	0.217 ± 0.09^{c}	3.71 ± 0.142^{d}	0.458 ± 0.018^{e}	6.57 ± 0.221^{f}	0.881 ± 0.326^{g}	2.14 ± 0.079^{h}
pHBA	1.07 ± 0.045^{a}	1.64 ± 0.076^{b}	1.45 ± 0.032^{c}	1.86 ± 0.057^{d}	0.749 ± 0.08^{e}	2.19 ± 0.061^{f}	2.91 ± 0.096^{g}	5.52 ± 0.234^{h}
Caffeic acid	0.568 ± 0.021^{a}	0.197 ± 0.007^{b}	5.60 ± 0.19^{c}	0.395 ± 0.013^{d}	6.43 ± 0.304^{e}	0.930 ± 0.031^{f}	0.306 ± 0.012^{g}	<LOD

Ferrulic acid	0.874 ± 0.039^{a}	1.20 ± 0.041^{b}	<LOD	0.619 ± 0.023^{c}	2.24 ± 0.100^{d}	0.182± 0.004^{e}	2.37 ± 0.074^{d}	<LOD
Protocatechuic acid	0.605 ± 0.027^{a}	2.22 ± 0.100^{b}	<LOD	<LOD	1.09 ± 0.042^{c}	6.12 ± 0.194^{d}	2.94 ± 0.082^{e}	<LOD
p-coumaric acid	0.195 ± 0.007^{a}	<LOD	<LOD	0.131 ± 0.003^{b}	0.614 ± 0.0271^{c}	<LOD	0.908 ± 0.035^{d}	<LOD
Gallic acid	0.253 ± 0.010^{a}	<LOD	<LOD	0.120 ± 0.003^{b}	0.07 ± 0.002^{c}	<LOD	0.155 ± 0.006^{d}	0.063 ± 0.002^{c}

*Results are expressed in mg/L as the mean ± SD of three independent repetitions. Different letters in each row means a difference at a significance level of p=0.05. LOD-limit of detection, the lowest concentration of the polyphenolic compound that can be detected by applied method. In our method it is 0.01 mg/L.
